# Translating Patient-Oriented Eczema Measure (POEM) scores into clinical practice by suggesting severity strata derived using anchor-based methods

**DOI:** 10.1111/bjd.12590

**Published:** 2013-12-02

**Authors:** CR Charman, AJ Venn, JC Ravenscroft, HC Williams

**Affiliations:** 1 Department of Dermatology, Royal Devon and Exeter HospitalExeter, U.K; 2 Division of Epidemiology and Public Health, University of NottinghamNottingham, U.K; 3 Centre of Evidence Based Dermatology, University of NottinghamNottingham, U.K

## Abstract

**Background** The Patient-Oriented Eczema Measure (POEM) is a validated, patient-derived assessment measure for monitoring atopic eczema severity, although further information on how different POEM scores translate into disease severity categories is needed for clinical trials, epidemiological research and audit.

**Objectives** We sought to determine the relationship between Patient-Oriented Eczema Measure (POEM) scores (range 0–28) and two Global Questions (GQ1 and 2) concerning patients’/parents’ views of the overall severity of their/their child's atopic eczema, in order to stratify POEM scores into five severity bands.

**Methods** POEM scores and GQs were completed by 300 patients from general practice and 700 patients from dermatology outpatient clinics, including 300 adults aged ≥ 16 years and 700 children.

**Results** The mean POEM score was 13·6 (range 0–28), and standard deviation (SD) was 7·2. Mean GQ1/GQ2 scores were 2·1/2·1, respectively (range 0–4 and SD 1·1 for both). The mean, mode and median of the GQ scores for each POEM score were used to devise possible POEM bandings. The proposed banding for POEM scores are: 0–2 (clear/almost clear); 3–7 (mild); 8–16 (moderate); 17–24 (severe); 25–28 (very severe), kappa coefficient 0·46.

**Conclusions** Severity banding of the POEM will allow more clinically meaningful use in everyday clinical practice and as a core outcome measure in future atopic eczema research.

**What's already known about this topic?**

- The POEM is a validated, reliable and simple tool for measuring atopic eczema severity in adults and children.
- POEM scores can range from 0 to 28, and have shown longitudinal sensitivity to change in the outpatient clinic and in clinical trials.

**What does this study add?**

- Using global patient-assessed anchor questions, POEM scores were categorized into five severity bands to improve interpretation in clinical practice and research. POEM scores of 0–2 = clear/almost clear, 3–7 = mild, 8–16 = moderate, 17–24 = severe, and 25–28 = very severe atopic eczema.

Advances in atopic eczema therapy depend on the availability of validated outcome measures which reflect disease severity in a way that is relevant to patients.^1^ The Patient-Oriented Eczema Measure (POEM) is a simple, valid, repeatable, and readily understandable tool for monitoring disease severity in children and adults with atopic eczema, which was originally developed to help readdress the imbalance between physician and patient-based outcome measures in eczema research (Fig. 1).^2^–^4^

**Figure 1:**
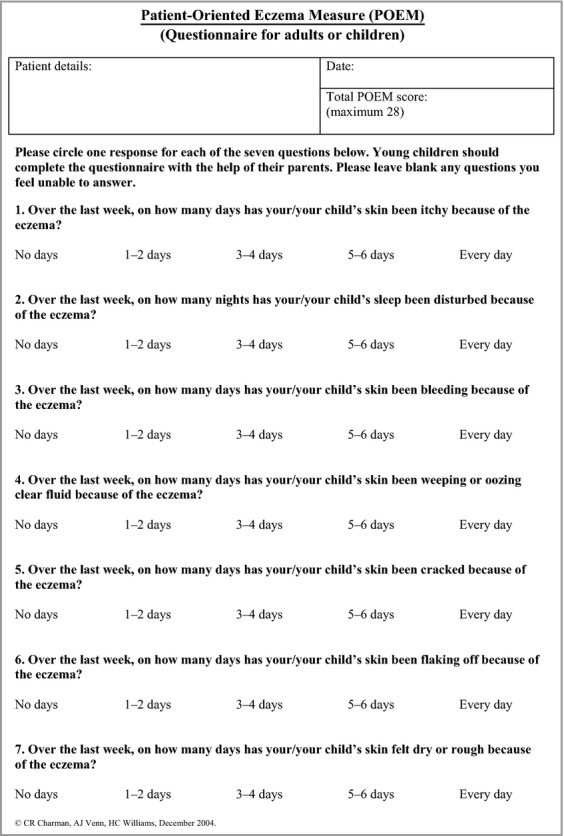
Patient-Oriented Eczema Measure (POEM), questionnaire for use in adults and children (or parents/carers) with atopic eczema.

The POEM has been widely recommended as an atopic eczema outcome measure in reviews and national guidelines,^5^–^8^ being suitable for use in the outpatient clinic, and for audit, epidemiological studies and clinical trials.^9^–^11^ Scoring sheets are available free on the U.K. Centre for Evidence Based Dermatology website (http://www.nottingham.ac.uk/scs/divisions/evidencebaseddermatology/resources/patientorientedeczemameasure.aspx). Linguistic translations are available on the Patient-Reported Outcome and Quality of Life Instruments Database (http://www.proqolid.org).

POEM scores improve as disease severity and quality of life improves, with one study suggesting a minimal clinically important difference (MCID) in POEM score of 3·4.^12^ However, further research is needed to provide information on the clinical meaning of individual scores, both for entry into clinical trials and outcome analysis.

The aim of this study was to explore the relationship between POEM scores and two global anchor questions concerning patients’ overall assessment of their disease severity, in order to establish a range of POEM scores corresponding to five different categories of disease severity.

## Patients and methods

**Figure 2:**
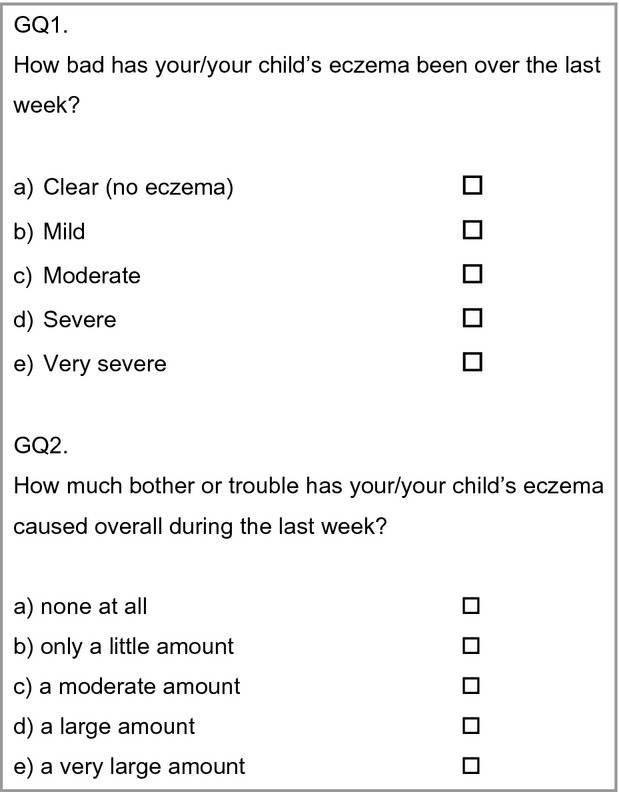
Global questions, GQ1 and 2

This was an open, prospective study of adult and paediatric patients with atopic eczema defined according to the U.K. Working Party's refinement of the Hanifin and Rajka diagnostic criteria, recruited from primary and secondary care.^13^ The POEM was used to measure atopic eczema severity against two global anchor questions (GQ1 and GQ2) relating to disease severity (Fig. 2). GQ1 was used as the primary outcome measure. Approval for the study was given by the local Research and Development departments.

It was estimated that 1000 questionnaires would be needed to categorize accurately the POEM scores into five bands, based on the normal distribution of POEM scores, and previous studies used to categorize patient-based scores using this method.^14^ In order to include patients from a diverse social and ethnic background, recruitment was carried out from two geographically distant U.K. dermatology outpatient departments (Royal Devon and Exeter Foundation Trust and Nottingham University Hospital NHS Trust) and six general practice surgeries in Devon, covering both urban and rural locations.

### Data processing and statistical analysis

Data were analysed using SPSS version 20 (IBM Corp., New York, NY, U.S.A). The distribution of each of the score variables was summarized using means and standard deviations. Medians and ranges were also computed because of the ordinal nature of these variables, and nonparametric methods were used to assess associations. For each POEM score the mean, mode and median GQ scores were used to group the POEM scores into possible severity strata (see Table3), and the kappa (κ) coefficient of agreement was calculated for each set of bands. A subset analysis was carried out on patients whose GQ1 scores disagreed with that predicted by two or more bands.

## Results

POEM scores and GQs were completed by 300 consecutive patients from general practice and 700 consecutive patients from dermatology outpatient clinics. The participants comprised 300 adults aged ≥ 16 years and 700 children (487 females and 513 males, median age 67 months, age range 1 month to 65 years, interquartile range 21 months to 17 years). The distribution of POEM and GQ scores are shown in Table 1, with slightly higher POEM scores seen in males (median 14 vs. 13, *P* = 0·01 for Mann–Whitney *U*-test) but no significant gender difference in GQ scores. POEM and GQ scores were significantly higher in patients recruited from secondary care (Mann–Whitney *P *< 0·001; Table 1), and on average were slightly higher in adults than children. The Spearman rank correlation coefficient showed a strong and highly statistically significant correlation between POEM scores and both the GQ1 (*r* = 0·78, *P *< 0·001) and GQ2 scores (*r *=* *0·77, *P *<* *0·001), which was not significantly affected by care setting, age or sex. There was a high correlation between GQ1 and GQ2 scores (Spearman *r *=* *0·82, *P *<* *0·001).

**Table 1:** Distribution of POEM and GQ scores overall and by care setting, sex and age

		POEM			GQ1			GQ2		
	*n*	Mean (SD)	Median (IQR)	*P*-value^a^	Mean (SD)	Median (IQR)	*P*-value^a^	Mean (SD)	Median (IQR)	*P*-value^a^
All patients	1000	13·60 (7·17)	13 (8–19)		2·10 (1·06)	2 (1–3)		2·09 (1·06)	2 (1–3)	
Care setting										
Primary	300	8·85 (5·27)	9 (4·25–13)	< 0·001	1·57 (0·94)	2 (1–2)	< 0·001	1·59 (0·96)	2 (1–2)	< 0·001
Secondary	700	15·63 (6·91)	16 (10–21)		2·32 (1·02)	2 (2–3)		2·31 (1·03)	2 (2–3)	
Sex										
Male	513	14·18 (7·44)	14 (8–20)	0·01	2·14 (1·04)	2 (1–3)	0·30	2·15 (1·10)	2 (1–3)	0·14
Female	487	12·98 (6·83)	13 (8–18)		2·05 (1·07)	2 (1–3)		2·04 (1·02)	2 (1–3)	
Age										
Child < 16	700	13·33 (7·39)	13 (8–19)	0·06	2·03 (1·09)	2 (1–3)	0·01	2·04 (1·09)	2 (1–3)	0·03
Adult	300	14·21 (6·58)	14 (10–19)		2·24 (0·96)	2 (2–3)		2·22 (0·97)	2 (2–3)	

IQR, interquartile range.

a P-values computed using Mann–Whitney *U*-test.

**Figure 3:**
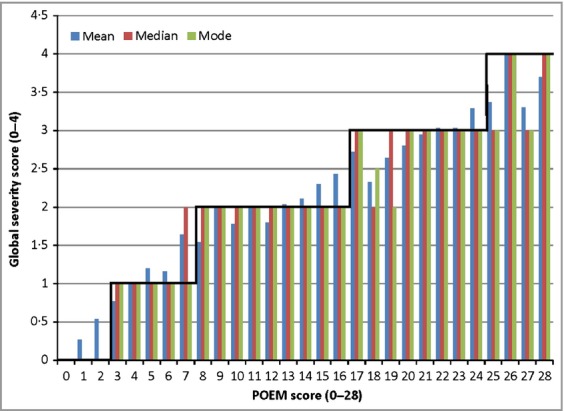
Relationship between the Patient-Oriented Eczema Measure (POEM) scores and the mode, mean and median of the Global Question (GQ1) score. The proposed banding scale of POEM scores 0–2, 3–7, 8–16, 17–24 and 25–28 is shown.

For each POEM score from 0 to 28, the distribution and the mean, mode and median of the corresponding GQ1 and GQ2 scores are shown in Table 2, with grey shaded areas illustrating POEM scores which could potentially have been included in either of the two adjacent bands. The two bandings with the highest ĸ values for GQ1 varied only in the positioning of POEM scores of 25 (Table 3). Banding option 2 showed almost as high a ĸ value as banding option 1, and also showed a higher ĸ value for GQ2. Of patients with POEM scores of 25, 53% rated their eczema as causing ‘a very large amount of bother’. Therefore banding option 2 was chosen as the final severity banding: POEM scores 0–2 = clear; 3–7 = mild; 8–16 = moderate; 17–24 = severe; 25–28 = very severe. Figure 3 illustrates the proposed POEM banding in relationship to the mean, mode and median of GQ1 scores.

**Table 2:** Number of patients with each POEM score and corresponding (a) GQ1 scores and (b) GQ2 scores (global patient-rated disease severity 0–4)

		GQ scores								
POEM score	Patient total	0	1	2	3	4	Mean	Median	Mode	Proposed banding
(a)										
0	16	16					0·00	0	0	Clear
1	22	16	6				0·27	0	0	
2	37	21	12	4			0·54	0	0	
3	26	6	20				0·77	1	1	Mild
4	19		19				1·00	1	1	
5	20	3	10	7			1·2	1	1	
6	56	2	43	11			1·16	1	1	
7	25		12	10	3		1·64	2	1	
8	54	8	13	29	4		1·54	2	2	Moderate
9	44		7	29	8		2·02	2	2	
10	45	4	6	31	4		1·78	2	2	
11	42		15	16	7	4	2·00	2	2	
12	40		12	24	4		1·80	2	2	
13	55		12	31	10	2	2·04	2	2	
14	36		1	30	5		2·11	2	2	
15	47		4	27	14	2	2·30	2	2	
16	47			27	20		2·43	2	2	
17	47			17	26	4	2·74	3	3	Severe
18	45		5	20	20		2·33	2	2/3	
19	47		1	21	19	6	2·64	3	2	
20	44		3	12	20	9	2·80	3	3	
21	21			4	14	3	2·95	3	3	
22	40			6	27	7	3·02	3	3	
23	33			2	28	3	3·03	3	3	
24	21				15	6	3·29	3	3	
25	30			1	17	12	3·37	3	3	
26	8					8	4·00	4	4	Very severe
27	10				7	3	3·30	3	3	
28	23				7	16	3·70	4	4	
(b)										
0	16	16					0	0	0	
1	22	16	6				0·27	0	0	Clear
2	37	12	19	6			0·84	1	1	
3	26	13	8	5			0·69	0·5	0	
4	19	2	17				0·89	1	1	Mild
5	20		1	7			1·35	1	1	
6	56	7	34	15			1·14	1	1	
7	25		14	9	2		1·52	1	1	
8	54		28	26			1·48	1	1	
9	44		7	31	6		1·98	2	2	Moderate
10	45		13	28	4		1·80	2	2	
11	42		9	32	1		1·81	2	2	
12	40		5	21	14		2·10	2	2	
13	55		6	35	12	2	2·18	2	2	
14	36		5	28	3		1·94	2	2	
15	47		8	27	10	2	2·13	2	2	
16	47	1	8	18	20		2·21	2	3	
17	47	1	2	13	27	4	2·66	3	3	
18	45		9	19	10	7	2·36	2	2	
19	47			21	16	10	2·77	3	2	Severe
20	44			17	16	11	2·86	3	2	
21	21			6	15		2·71	3	3	
22	40			8	24	8	3·00	3	3	
23	33				33		3·00	3	3	
24	21				13	8	3·38	3	3	
25	30				14	16	3·53	4	4	
26	8				5	3	3·38	3	3	
27	10					10	4	4	4	Very severe
28	23				8	15	3·65	4	4	

Grey shaded scores represent two possible bandings based on mean, median and mode values and POEM subscore analysis.

**Table 3:** Kappa coefficients of agreement for different proposed sets of POEM severity bands, with final severity band highlighted

Possible POEM bandings					K coefficient of agreement	
					GQ1	GQ2
Clear	Mild	Moderate	Severe	Very severe	Patient global severity	Patient global bother
0–2	3–7	8–16	17–25	26–28	0.466	0.407
0–2	3–7	8–16	17–24	25–28	0.463	0.414
0–2	3–6	7–16	17–25	26–28	0.460	0.397
0–1	2–7	8–16	17–25	26–28	0.450	0.413
0–1	2–7	8–16	17–24	25–28	0.447	0.419
0–1	2–6	7–16	17–24	25–28	0.442	0.409
0–1	2–7	8–16	17–26	27–28	0.438	0.415

### Subgroup analysis

#### Overview of POEM scores falling outside proposed banding

No patients showed a GQ1 score > 2 points outside of that predicted by the final POEM severity bands. There were 22 patients (2·2%) whose actual GQ1 score was two points lower than the final severity banding would have predicted from their POEM score, although this figure fell to only two patients (0·2%) when using the GQ2 score. There were 15 patients (1·5%) whose actual GQ1 score was two points higher than the final severity banding would have predicted, although again this figure fell to only nine patients (0·9%) when patients’ GQ2 scores were used as a measure of disease severity.

#### POEM scores in 0–2 (clear) category

Table 2 illustrates that although 53 of 75 (71%) of patients in the clear category self-rated their eczema as ‘clear’, a further 18 of 75 (24%) and 4 of 75 (5%) patients rated their eczema as mild or moderate, respectively. Dryness and itching were the most common symptoms reported by patients in this severity band, with no patients reporting symptoms of acute eczema such as bleeding or weeping. In view of the difficulties in precisely defining eczema as ‘clear’, the term ‘clear or almost clear’ was used to define this category.

#### POEM scores of 25

As the two bandings with the highest ĸ values for GQ1 varied only in the positioning of POEM scores of 25 in either the severe or very severe category, the individual symptom scores of these patients were examined, to justify inclusion in the ‘very severe’ banding. All the patients with POEM scores of 25 reported daily itching and sleep loss of ≥ 5 nights a week, and 93% reported bleeding ≥ 5 days a week.

## Discussion

Currently the POEM is recommended as one of the three most adequately validated and tested outcome measures for atopic eczema, alongside the SCORAD index and the Eczema Area and Severity Index (EASI).^1^,^6^,^15^,^16^ Of these three outcome measures, the POEM is the only measure which is fully patient-derived and patient-assessed. All three outcome measures provide complementary information on disease severity.^1^,^6^,^17^

The inclusion of two global patient-rated questions provided a more accurate measure of eczema severity. Self-assessment of eczema may be limited by personal experience, or influenced by comparison with families or friends with the condition. Similarly, the degree of bother caused by the eczema may be influenced by external factors at home, school or work. The assessment of ‘bother’ (as in GQ2) has been used successfully in asthma outcome measure research, and was easily understood by patients in the development of the POEM.^2^

Overall < 4% of patients’ POEM scores fell outside the proposed banding by two bands. The anonymous design of this study did not allow more detailed analysis of factors contributing to these outlying scores, although it is recognized that patients with associated ichthyosis may score highly on domains such as dryness or roughness and flaking without significant symptoms of eczema. Patients with low POEM scores but high GQ scores may reflect less familiarity with the disease, or worries about prognosis or treatment.

In primary care the POEM bands defined by this research could be used to support the decision to refer to secondary care (e.g. in children experiencing 1–2 weeks of flares a month), or to guide primary care physicians in appropriate prescribing of topical steroid therapy, with POEM scores of 8–16 or ≥ 17 supporting the need for moderately potent or potent topical steroids, respectively.^8^ The POEM severity banding may also provide a useful decision-making tool for primary care physicians considering topical calcineurin inhibitor therapy for patients with moderate or severe atopic eczema (POEM scores of ≥ 8).^8^

Recent atopic eczema research has focused on the development of consensus-based sets of core outcome domains for atopic eczema, for use in controlled trials and clinical record keeping.^1^ The Harmonizing Outcomes Measures for Eczema (HOME) initiative has identified four core outcomes which are recommended for inclusion in all future atopic eczema trials in order to enhance clinical interpretability and to enable meta-analyses across different studies: patient symptoms, physician-assessed clinical signs, quality of life, and a measurement for long-term control of flares.^1^ The POEM stratification proposed in this study offers researchers a tool with which to capture longitudinal patient symptoms, and long-term control of flares, with POEM scores of ≥ 8 and ≥ 16 representing moderate to severe flares, respectively, and POEM scores of ≤ 2 representing eczema in remission. It is hoped that the POEM will be considered as a core outcome measure for future atopic eczema clinical trials, with the final severity bands providing an accurate and easily interpretable patient-based quantitative measure of long-term disease control.

## References

[b1] Schmitt J, Spuls P, Boers M (2012). Towards global consensus on outcome measures for atopic eczema research: results of the HOME II meeting. Allergy.

[b2] Charman CR, Venn AJ, Williams HC (2004). The Patient-Oriented Eczema Measure: development and initial validation of a new tool for measuring atopic eczema severity from the patients’ perspective. Arch Dermatol.

[b3] Charman CR, Williams HC (2000). Outcome measures of disease severity in atopic eczema. Arch Dermatol.

[b4] Charman CR, Chambers C, Williams HC (2003). Measuring atopic dermatitis severity in randomised controlled clinical trials: what exactly are we measuring?. J Invest Dermatol.

[b5] Ricci G, Dondi A, Patrizi A (2009). Useful tools for the management of atopic dermatitis. Am J Clin Dermatol.

[b6] Schmitt J, Langan S, Williams HC (2007). What are the best outcome measurements for atopic eczema? A systematic review. J Allergy Clin Immunol.

[b7] Williams HC, Grindlay DJC (2009). What's new in atopic eczema? An analysis of systematic reviews published in 2007 and 2008. Part 1. Definitions, causes and consequences of eczema. Clin Exp Dermatol.

[b8] National Institute for Health and Clinical Excellence (2007). http://guidance.nice.org/CG57.

[b9] Thomas KS, Koller K, Dean T (2011). A multicentre randomized controlled trial of ion-exchange water softeners for the treatment of eczema in children: the Softened Water Eczema Trial (SWET). Health Technol Assess.

[b10] Schram ME, Roekevisch E, Leeflang MM (2011). A randomized trial of methotrexate versus azathioprine for severe atopic eczema. J Allergy Clin Immunol.

[b11] Armstrong AW, Kim RH, Idriss NZ (2011). Online video improves clinical outcomes in adults with atopic dermatitis: a randomized controlled trial. J Am Acad Dermatol.

[b12] Schram ME, Spuls PI, Leeflang MMG (2012). EASI, (objective) SCORAD and POEM for atopic eczema: responsiveness and minimal clinically important difference. Allergy.

[b13] Williams HC, Burney PG, Hay RJ (1994). The UK Working Party's diagnostic criteria for atopic dermatitis. Br J Dermatol.

[b14] Hongbo Y, Thomas CL, Harrison MA (2005). Translating the science of quality of life into practice: what do dermatology life quality index scores mean?. J Invest Dermatol.

[b15] European Task Force on Atopic Dermatitis (1993). Severity scoring of atopic dermatitis: the SCORAD index. Dermatology.

[b16] Tofte S, Graeber M, Cherill R (1998). Eczema area and severity index (EASI): a new tool to evaluate atopic dermatitis. J Eur Acad Dermatol Venereol.

[b17] Charman CR, Venn AJ, Williams HC (2005). Measuring atopic eczema severity visually; which variables are most important to patients?. Arch Dermatol.

